# As good as human experts in detecting plant roots in minirhizotron images but efficient and reproducible: the convolutional neural network “RootDetector”

**DOI:** 10.1038/s41598-023-28400-x

**Published:** 2023-01-25

**Authors:** Bo Peters, Gesche Blume-Werry, Alexander Gillert, Sarah Schwieger, Uwe Freiherr von Lukas, Juergen Kreyling

**Affiliations:** 1 Experimental Plant Ecology, Institute of Botany and Landscape Ecology, University of Greifswald, Soldmannstraße 15, 19489 Greifswald, Germany; 2 Department of Ecology and Environmental Science, Umeå University, Umeå, Sweden; 3 Fraunhofer Institute for Computer Graphics Research IGD, Rostock, Germany; 4 Institute for Visual & Analytic Computing, University of Rostock, Rostock, Germany

**Keywords:** Imaging, Ecosystem ecology

## Abstract

Plant roots influence many ecological and biogeochemical processes, such as carbon, water and nutrient cycling. Because of difficult accessibility, knowledge on plant root growth dynamics in field conditions, however, is fragmentary at best. Minirhizotrons, i.e. transparent tubes placed in the substrate into which specialized cameras or circular scanners are inserted, facilitate the capture of high-resolution images of root dynamics at the soil-tube interface with little to no disturbance after the initial installation. Their use, especially in field studies with multiple species and heterogeneous substrates, though, is limited by the amount of work that subsequent manual tracing of roots in the images requires. Furthermore, the reproducibility and objectivity of manual root detection is questionable. Here, we use a Convolutional Neural Network (CNN) for the automatic detection of roots in minirhizotron images and compare the performance of our RootDetector with human analysts with different levels of expertise. Our minirhizotron data come from various wetlands on organic soils, i.e. highly heterogeneous substrates consisting of dead plant material, often times mainly roots, in various degrees of decomposition. This may be seen as one of the most challenging soil types for root segmentation in minirhizotron images. RootDetector showed a high capability to correctly segment root pixels in minirhizotron images from field observations (F1 = 0.6044; r^2^ compared to a human expert = 0.99). Reproducibility among humans, however, depended strongly on expertise level, with novices showing drastic variation among individual analysts and annotating on average more than 13-times higher root length/cm^2^ per image compared to expert analysts. CNNs such as RootDetector provide a reliable and efficient method for the detection of roots and root length in minirhizotron images even from challenging field conditions. Analyses with RootDetector thus save resources, are reproducible and objective, and are as accurate as manual analyses performed by human experts.

## Introduction

Quantifying and monitoring biomass accumulation from plants is of growing interest for many scientific fields as it provides a valuable metric for complex ecosystem dynamics. Around 30–95% of plant biomass is located belowground across biomes in form of roots^1^, and roots mediate the carbon input into the soil through rhizodeposition^2^. Thus, knowledge about root growth dynamics, i.e. spacial and temporal differences in growth, longevity and turnover, is crucial for the understanding of carbon stocks and fluxes in ecosystems, and their representation is essential in coupled biosphere–atmosphere models^3,4^. However, even basic data such as root length, density, seasonal activity or growth rates are fragmentary at best due to difficult accessibility and high susceptibility of roots to disturbance^5^. Many methods for surveys of root growth dynamics under field conditions are not very accurate and highly destructive. The washing out of soil samples, for example, has been shown to record only 60% of the biomass, as fine roots, functionally the most important root type, are commonly lost^6^. Destructive methods also do not allow for insights into root growth dynamics over time, as they provide a mere snapshot. Therefore, by far the most important tool for recording root growth dynamics in the field has become the so-called minirhizotron technique^7^. Minirhizotrons are transparent tubes, which, once inserted into the soil, enable regular recordings of root growth at the tube-soil interface via imaging by circular scanners or cameras. As this method of sampling is non-destructive and minimally invasive, it can be conducted as often as required and over any length of time, thus enabling precise measurement and visualization of important root growth parameters such as initiation of growth, elongation and increase in diameter as well as turnover. Therefore, the use of minirhizotrons is a highly effective method for detailed investigation even of the finest and most short-lived root types (lifespans of days to weeks) in high temporal and spatial resolution, allowing for investigations of ever more apparent decoupled belowground and aboveground seasonal growth patterns (‘phenology’)^8–10^. Until now, the detection of roots in the minirhizotron images is done manually by human analysts, at least in field studies dealing with heterogeneous substrates and multiple species. Depending on image quality and root abundance, the processing of a single image can take several hours. This is one reason why there are very few long-term measurement series in fine temporal resolution on root growth dynamics. Moreover, the high time demand does not allow for the quantification of spatial variation in root traits and root growth dynamics^11^. This shows that the biggest obstacle for providing sound, temporally and spatially highly resolved data on root growth dynamics is the arduous detection of roots in complex minirhizotron images.

The recent development of Convolutional Neural Networks (CNN) has sparked interest due to their capacity to automatically extract relevant features directly from images without the need for human feature design. CNNs have been shown to outperform traditional algorithms in most computer vision tasks^12^ and as such provide a powerful, inexpensive and time-saving new method for (semi-)automatic analysis of minirhizotron images. Indeed, first attempts under ideal conditions with relatively homogeneous substrate and young roots of single species are promising^13–15^. Another advantage of using CNNs over manual segmentation is their improved objectivity and repeatability in comparison to human analysts. As long as the conditions and image quality are relatively constant (e.g., illumination, contrast), the accuracy of automatic feature recognition using CNNs is constant as well. In contrast, accuracy of human analysts may vary greatly depending on state of mind (e.g., fatigue, time pressure) and even more so between individuals.

Here, we introduce RootDetector, a Convolutional Neural Network-based approach for classifying roots and extracting metrics for root length in minirhizotron images from field studies. We trained RootDetector with data from a mesocosm experiment and a field experiment that included different organic soils and a variety of plant species. We compared RootDetector’s performance with that of human analysts and investigated how differences in experience with plant physiology and digital root measuring tools between groups of human analysts (novice, advanced, expert) affect the accuracy of manual root segmentation. We furthermore validated RootDetector’s ability to classify root pixels and to quantify root length on randomly selected minirhizotron images from the field.

## Material and methods

### Datasets

#### Image acquisition

For this study, we assembled three datasets: one for training of the RootDetector Convolutional Neural Network (Training-Set), one for a performance comparison between humans and RootDetector in segmenting roots in minirhizotron images (Comparison-Set), and one for the validation of the algorithm (Validation-Set). The Training-Set contained 129 images comprised of 17 randomly selected minirhizotron images sampled in a mesocosm experiment (see “Mesocosm sampling” Section), 47 randomly selected minirhizotron images sampled in a field study (see “Field sampling” Section) as well as the 65 minirhizotron images of soy roots published by Wang et al.^15^. The Comparison-Set contained 25 randomly selected minirhizotron images from the field-study which all were not part of the images included in the Training- and Validation-Sets. The Validation-Set contained 10 randomly selected minirhizotron images from the same field study, which had not been used in the Training-Set. All images were recorded with 2550 ✕ 2273 pixels at 300 dpi with a CI-600 In-Situ Root Imager (CID Bio-Science Inc., Camas, WA, USA) and stored as .tiff files to reduce compression loss. For all training and evaluation purposes we used raw, unprocessed output images from the CI-600.

#### Mesocosm sampling

The mesocosm experiment was established in 2018 on the premises of the Institute for Botany and Landscape Ecology of the University of Greifswald (Fig. S1). It features 108 heavy duty plastic buckets of 100 l each, filled to two thirds of their height with moderately decomposed sedge fen peat. Each mesocosm contained one minirhizotron (inner diameter: 64 mm, outer diameter: 70 mm, length: 650 mm) installed at a 45°angle and capped in order to avoid penetration by light. The mesocosms were planted with varying compositions of plant species that typically occur in north-east German sedge fens (*Carex rostrata, Carex acutiformis, Glyceria maxima, Equisetum fluviatile, Juncus inflexus, Mentha aquatica, Acorus calamus* and *Lycopus europaeus*). The mesocosms were subjected to three different water table regimes: stable at soil surface level, stable at 20 cm below soil surface and fluctuating between the two levels every two weeks. The minirhizotrons were scanned weekly at two levels of soil depth (0–20 cm and 15–35 cm) between April 2019 and December 2021, resulting in roughly 9500 minirhizotron images of 216 × 196 mm. Manual quantification of root length would, based on own experience, take approximately three hours per image, resulting in approximately 28,500 h of manual processing for the complete dataset. Specimens planted were identified by author Dr. Blume-Werry, however no voucher specimen were deposited. All methods were carried out in accordance with relevant institutional, national, and international guidelines and legislation.

#### Field sampling

The field study was established as part of the Wetscapes project in 2017^16^. The study sites were located in Mecklenburg-Vorpommern, Germany, in three of the most common wetland types of the region: alder forest, percolation fen and coastal fen (Fig. S2). For each wetland type, a pair of drained versus rewetted study sites was established. A detailed description of the study sites and the experimental setup can be found in Jurasinski et al.^16^. At each site, 15 minirhizotrons (same diameter as above, length: 1500 mm) were installed at 45° angle along a central boardwalk. The minirhizotrons have been scanned biweekly since April 2018, then monthly since January 2019 at two to four levels of soil depth (0–20 cm, 20–40 cm, 40–60 cm and 60–80 cm), resulting in roughly 12,000 minirhizotron images of 216 × 196 cm, i.e. an estimated 36,000 h of manual processing for the complete dataset. Permission for the study was obtained from the all field owners.

**Figure 1:**
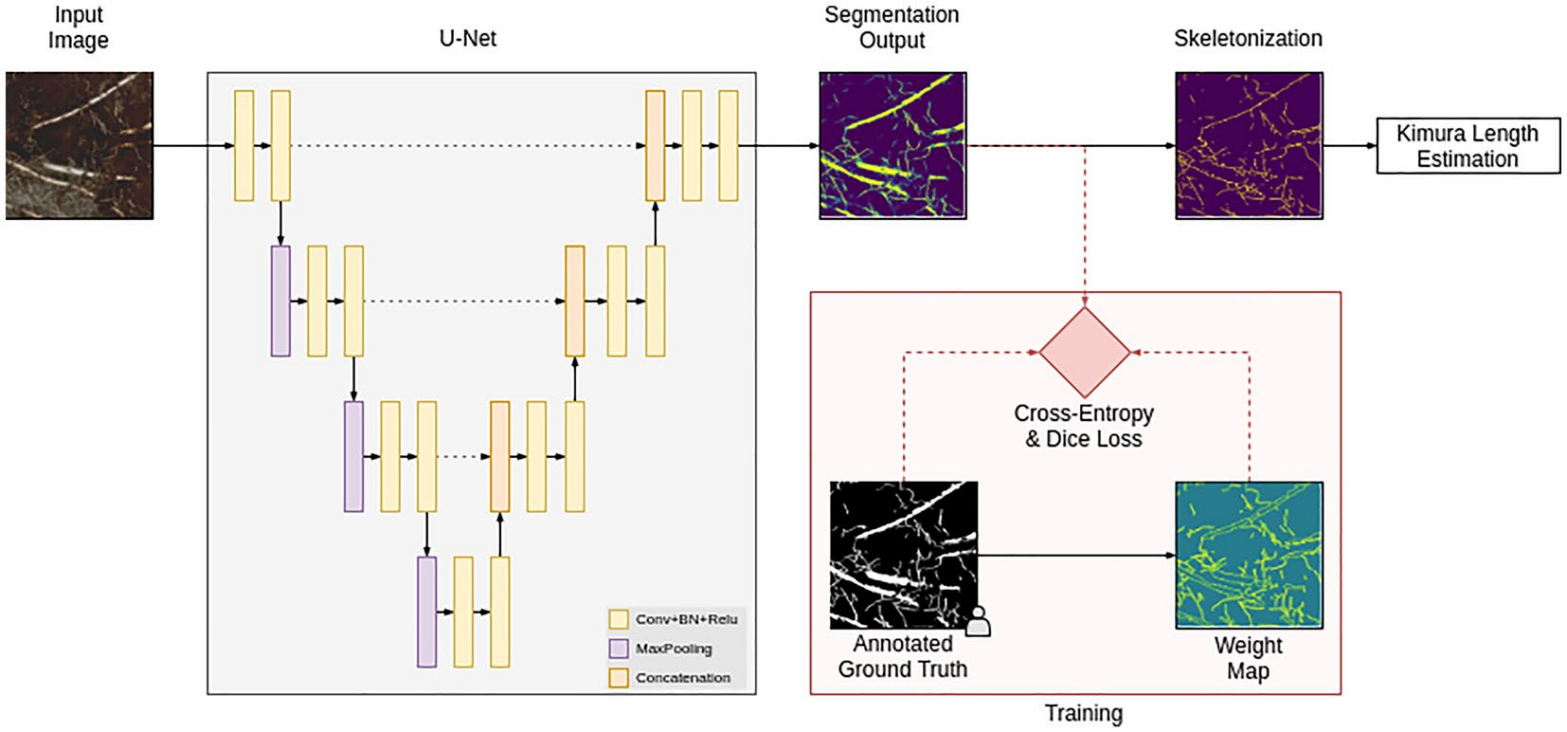
Overview of the RootDetector system. The main component is a semantic segmentation network based on the U-Net architecture. The root length is estimated by skeletonizing the segmentation output and applying the formula introduced by Kimura et al.^17^. During training only, a weight map puts more emphasis on fine roots.

### The CNN RootDetector

#### Image annotation

**Figure 2:**
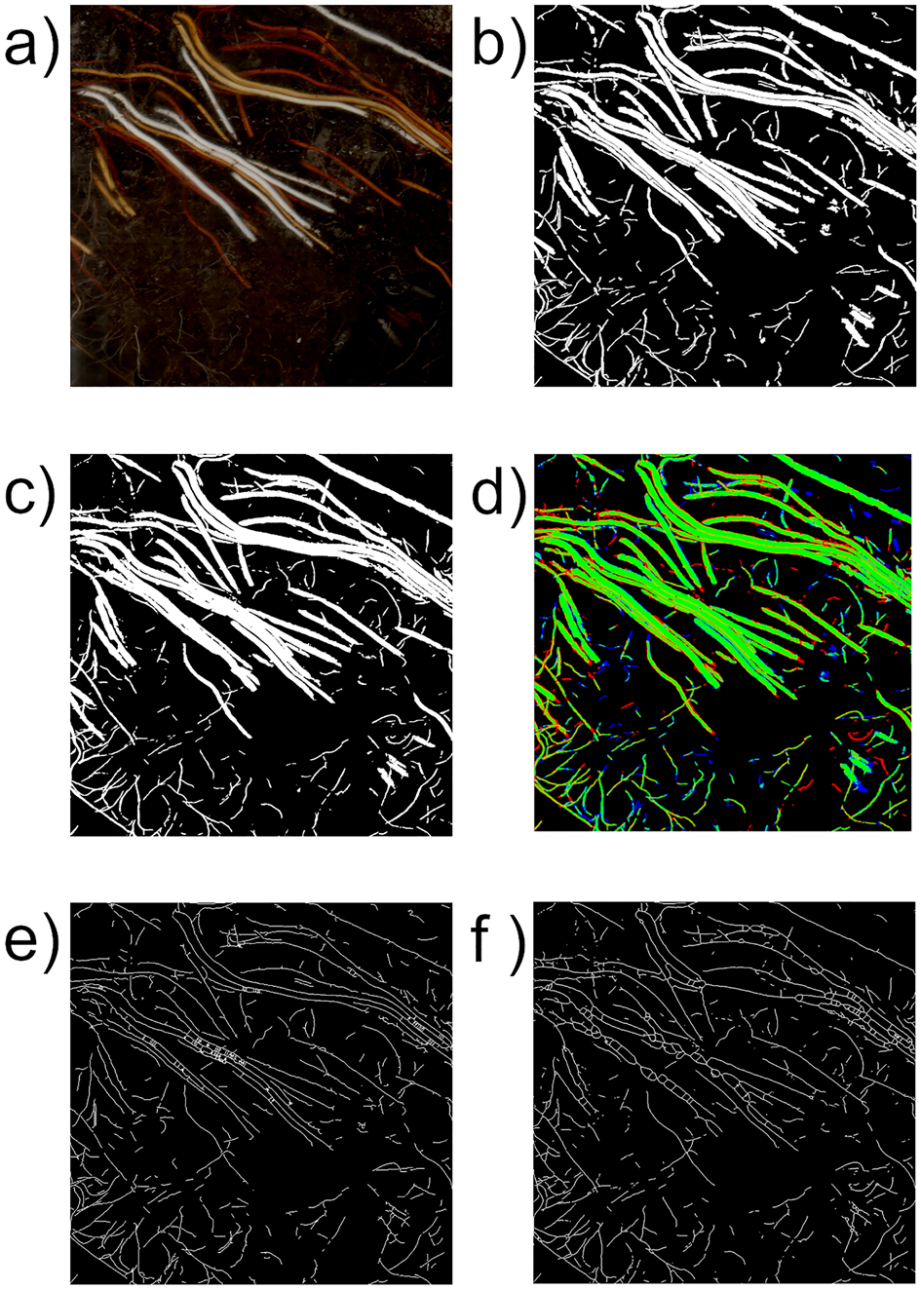
Example of segmentation and result of skeletonization. A 1000 by 1000 pixel input image (**a**), the manually annotated ground truth image (**b**), the RootDetector estimation image (**c**), the combined representation image (error map, d with green indicating true positives, red indicating false positive, blue indicating false negatives), the skeletonized RootDetector estimation image (**e**), and the skeletonized ground truth image (**f**).

For the generation of training data for the CNN, human analysts manually masked all root pixels in the 74 images of the Training-Set using GIMP 2.10.12. The resulting ground truth data are binary, black-and-white images in Portable Network Graphics (.png) format, where white pixels represent root structures and black pixels represent non-root objects and soil (Fig. 2b). All training data were checked and, if required, corrected by an expert (see “Selection of participants” for definition). The Validation-Set was created in the same way but exclusively by experts.

#### Architecture

RootDetector's core consists of a Deep Neural Network (DNN) based on the U-Net image segmentation architecture[27]nd is implemented in TensorFlow and Keras frameworks^18^. Although U-Net was originally developed for biomedical applications, it has since been successfully applied to other domains due to its generic design.

RootDetector is built up of four down-sampling blocks, four up-sampling blocks and a final output block (Fig. 1). Every block contains two 3 × 3 convolutional layers, each followed by rectified linear units (ReLU). The last output layer instead utilizes Sigmoid activation. Starting from initial 64 feature channels, this number is doubled in every down-block and the resolution is halved via 2 × 2 max-pooling. Every up-block again doubles the resolution via bilinear interpolation and a 1 × 1 convolution which halves the number of channels. Importantly, after each up-sampling step, the feature map is concatenated with the corresponding feature map from the down-sampling path. This is crucial to preserve fine spatial details.

Our modifications from the original architecture include BatchNormalization^19^ after each convolutional layer which significantly helps to speed up the training process and zero-padding instead of cropping as suggested by Ronneberger, Fischer, & Brox^20^ to preserve the original image size.

In addition to the root segmentation network, we trained a second network to detect foreign objects, specifically the adhesive tape that is used as a light barrier on the aboveground part of the minirhizotrons. We used the same network architecture as above and trained in a supervised fashion with the binary cross-entropy loss. During inference, the result is thresholded (predefined threshold value: 0.5) and used without post-processing.

#### Training

We pre-trained RootDetector on the COCO dataset^21^ to generate a starting point. Although the COCO dataset contains a wide variety of image types and classes not specifically related to minirhizotron images, Majurski et al.^22^ showed, that for small annotation counts, transfer-learning even from unrelated datasets may improve a CNNs performance by up to 20%. We fine-tuned for our dataset with the Adam optimizer^23^ for 15 epochs and trained on a total of 129 images from the Training-Set (17 mesocosm images, 47 field-experiment images, 65 soy root images). To enhance the dataset size and reduce over-fitting effects, we performed a series of augmentation operations as described by Shorten & Khoshgoftaar^24^. In many images, relatively coarse roots (> 3 mm) occupied a major part of the positive (white) pixel space, which might have caused RootDetector to underestimate fine root details overall. Similarly, negative space (black pixels) between tightly packed, parallel roots was often very small and might have impacted the training process to a lesser extent when compared to large areas with few or no roots (Fig. 2). To mitigate both effects, we multiplied the result of the cross-entropy loss map with a weight map which emphasizes positive–negative transitions. This weight map is generated by applying the following formula to the annotated ground truth images:

1 $$\omega \left( x \right) = 1 - \left( {\tanh \left( {2\tilde{x} - 1} \right)} \right)^{2}$$

where ω(x) is the average pixel value of the annotated weight map in a 5 × 5 neighborhood around pixel x. Ronneberger, Fischer, & Brox^20^ implemented a similar weight map, however with stronger emphasis on space between objects. As this requires computation of distances between two comparatively large sets of points, we adapted and simplified their formula to be computable in a single 5 × 5 convolution.

For the loss function we applied a combination of cross-entropy and Dice loss ^25^:

2 $${\mathcal{L}} = {\mathcal{L}}_{CE} + \lambda {\mathcal{L}}_{Dice} = - \frac{1}{N}\sum\nolimits_{i} {w\left( {x_{i} } \right)y_{i} \log \left( {x_{i} } \right) + \lambda \frac{{2\sum\nolimits_{i} {x_{i} y_{i} } }}{{\sum\nolimits_{i} {x_{i}^{2} \sum\nolimits_{i} {y_{i}^{2} } } }}}$$

where x are the predicted pixels, y the corresponding ground truth labels, N the number of pixels in an image and λ a balancing factor which we set to 0.01. This value was derived empirically. The Dice loss is applied per-image to counteract the usually high positive-to-negative pixel imbalance. Since this may produce overly confident outputs and restrict the application of weight maps, we used a relatively low value for λ.

#### Output and post-processing

RootDetector generates two types of output. The first type of output are greyscale .png files in which white pixels represent pixels associated with root structures and black pixels represent non-root structures and soil (Fig. 2c). The advantage of .png images is their lossless ad artifact-free compression at relatively small file sizes. RootDetector further skeletonizes the output images and reduces root-structures to single-pixel representations using the skeletonize function of scikit-image v. 0.17.1 (^26^; Fig. 2e,f). This helps to reduce the impact of large diameter roots or root-like structures such as rhizomes in subsequent analyses and is directly comparable to estimations of root length. The second type of output is a Comma-separated values (.csv) file, with numerical values indicating the number of identified root pixels, the number of root pixels after skeletonization, the number of orthogonal and diagonal connections between pixels after skeletonization and an estimation of the physical combined length of all roots for each processed image. The latter is a metric commonly used in root research as in many species, fine roots provide most vital functions such as nutrient and water transport^3^. Therefore, the combined length of all roots in a given space puts an emphasis on fine roots as they typically occupy a relatively smaller fraction of the area in a 2D image compared to often much thicker coarse roots. To derive physical length estimates from skeletonized images, RootDetector counts orthogonal- and diagonal connections between pixels of skeletonized images and employs the formula proposed by Kimura et al.^17^ (Eq. 3).

3 $$L = \left[ {N_{d}^{2} + \left( {N_{d} + N_{o} /2} \right)^{2} } \right]^{{1/2}} + N_{o} /2$$

where N_d_ is the number of diagonally connected and N_o_ the number of orthogonally connected skeleton pixels. To compute N_d_ we convolve the skeletonized image with two 2 × 2 binary kernels, one for top-left-to-bottom-right connections and another for bottom-left-to-top-right connections and count the number of pixels with maximum response in the convolution result. Similarly, N_o_ is computed with a 1 × 2 and a 2 × 1 convolutional kernels.

### Performance comparison

#### Selection of participants

For the performance comparison, we selected 10 human analysts and divided them into three groups of different expertise levels in plant physiology and with the usage of digital root measuring tools. The novice group consisted of 3 ecology students (2 bachelor's, 1 master's) who had taken or were taking courses in plant physiology but had no prior experience with minirhizotron images or digital root measuring tools. This group represents undergraduate students producing data for a Bachelor thesis or student assistants employed to process data. The advanced group consisted of 3 ecology students (1 bachelor's, 2 master's) who had already taken courses in plant physiology and had at least 100 h of experience with minirhizotron images and digital root measuring tools. The expert group consisted of 4 scientists (2 PhD, 2 PhD candidates) who had extensive experience in root science and at least 250 h of experience with digital root measuring tools. All methods were carried out in accordance with relevant institutional, national, and international guidelines and legislation and informed consent was obtained from all participants.

#### Instruction and root tracing

All three groups were instructed by showing them a 60 min live demo of an expert tracing roots in minirhizotron images, during which commonly encountered challenges and pitfalls were thoroughly discussed. Additionally, all participants were provided with a previously generated, in-depth manual containing guidelines on the identification of root structures, the correct operation of the root tracing program and examples of often encountered challenges and suggested solutions. Before working on the Comparison-Set, all participants traced roots in one smaller-size sample image and received feedback from one expert.

#### Image preparation and root tracing

Because the minirhizotron images acquired in the field covered a variety of different substrates, roots of different plant species, variance in image quality, and because tracing roots is very time consuming, we decided to maximize the number of images by tracing roots only in small sections, in order to cover the largest number of cases possible. To do this, we placed a box of 1000 × 1000 pixels (8.47 × 8.47 cm) at a random location in each of the images in the Comparison-Set and instructed participants to trace only roots within that box. Similarly, we provided RootDetector images where the parts of the image outside the rectangle were occluded. All groups used RootSnap! 1.3.2.25 (CID Bio-Science Inc., Camas, WA, USA;^27^), a vector based tool to manually trace roots in each of the 25 images in the comparison set. We decided on RootSnap! due to our previous good experience with the software and its' relative ease of use. The combined length of all roots was then exported as a csv file for each person and image and compared to RootDetector’s output of the Kimura root length.

### Validation

We tested the accuracy of RootDetector on a set of 10 image segments of 1000 by 1000 pixels cropped from random locations of the 10 images of the Validation-Set. These images were annotated by a human expert without knowledge of the estimations by the algorithm and were exempted from the training process. As commonly applied in binary classification, we use the F1 score as a metric to evaluate the performance RootDetector. F1 is calculated from precision (Eq. 4) and recall (Eq. 5) and represents their harmonic mean (Eq. 6). Ranging from 0 to 1, higher values indicate high classification (segmentation) performance. As one of the 10 image sections contained no roots and thus no F1 Score was calculable, it was excluded from the validation. We calculated the F1 score for each of the nine remaining image sections and averaged the values as a metric for overall segmentation performance.

4 $$Precision\;(P) = \frac{{tp}}{{tp + fp}}$$

5 $$Recall\;(R) = \frac{{tp}}{{tp + fn}}$$

6 $$F1 = 2*\frac{{P*R}}{{P + R}}$$

where P = precision, R = recall, tp = true positives; fp = false positives, fn = false negatives.

### Statistical analysis

We used R Version 4.1.2 (R Core Team, 2021) for all statistical analyses and R package ggplot2 Version 3.2.1^28^ for visualizations. Pixel identification-performance comparisons were based on least-squares fit and the Pearson method. Root length estimation-performance comparisons between groups of human analysts (novice, advanced, expert) and RootDetector were based on the respective estimates of total root length plotted over the minirhizotron images in increasing order of total root length. Linear models were calculated using the lm function for each group of analysts. To determine significant differences between the groups and the algorithm, 95% CIs as well as 83% CIs were displayed and RootDetector root length outside the 95% CI were considered significantly different from the group estimate at α = 0.05^29^. The groups of human analysts were considered significantly different if their 83% CIs did not overlap, as the comparison of two 83% CIs approximates an alpha level of 5%^30,31^.

This study is approved by Ethikkommission der Universitätsmedizin Greifswald, University of Greifswald, Germany.

## Results

### Performance comparison

**Figure 3:**
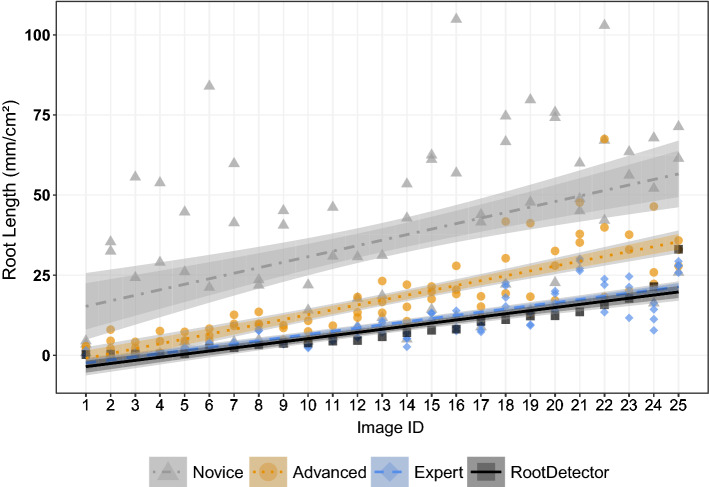
The CNN RootDetector performs as good as human experts (analysts with scientific background in root ecology and > 250 h experience in annotating roots), as indicated by the linear regression of RootDetector lying within the 95% CI (darker shaded ribbons) of the expert group. Groups of human analysts differ in their estimation of root lengths, as indicated by non-overlapping 83% CIs (lighter shaded ribbons). Novices had theoretical input on root ecology but no experience with minirhizotron images, advanced analysts had theoretical input on root ecology and > 100 h of experience with annotating minirhizotron images. Images are sorted by increasing root length according to RootDetector along the x-axis. The regression coefficient was 0.23 for the novice group; 0.67 for the advanced group, 0.74 for the expert group and 0.82 for RootDetector.

Human analysts differed strongly in total root length annotated per minirhizotron image section (Fig. 3). Novice participants generally estimated highest root length, while experts found the lowest total root length. Novices estimated on average 1324% (SD 2508%), and advanced participants on average 320% (SD 342%) of total root length(mm) as compared to the expert group. RootDetector estimated on average 87% (SD 38%) of total root length (mm) compared to experts. Variation in total root length estimation, as expressed by the 95% CI in Fig. 3, was highest between the three novices and lowest between the four experts. Root length quantification by RootDetector was indifferent from the expert group, but lower than the novice and advanced groups.

### Overall performance of RootDetector

**Figure 4:**
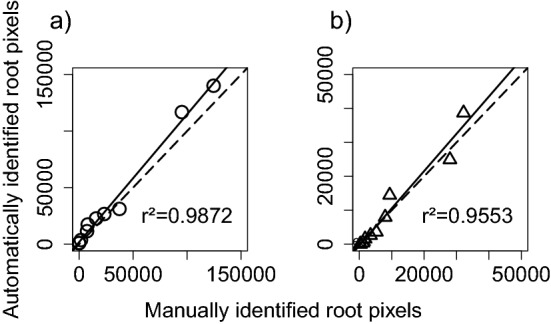
Correlation between (**a**) total root pixels and (**b**) total root pixels after skeletonization as detected by the CNN RootDetector and expert human analysis based on ten 1000 × 1000 pixel (8.47 cm × 8.47 cm) image segments cropped from randomly selected minirhizotron images from the field study (Validation-Set). Dotted lines represent the 1:1 line, solid lines the least-squares correlation.

RootDetector showed a high capability to correctly segment roots in the Validation-Set of minirhizotron images not used during the training phase. Total root pixels in the images were detected with a F1 score of 0.6044. The correlation between total number of root pixels detected by RootDetector and, independently, by one human expert was very high (r^2^ = 0.99) and with uniform residuals across the data range (Fig. 4a). The correlation was also high after skeletonization (r^2^ = 0.96; Fig. 4b).

### Discussion

RootDetector provides the general advantage of perfect reproducibility and objectivity, two points that are questionable at best for human analysts^32^. Our study clearly showed that there was large variation in root length estimates with novices annotating almost 3-times higher root length/cm^2^ per image compared to expert analysts (Fig. 3). Interestingly, this variation shrank for more experienced analysts, i.e., the more time analysts have already spent on analysing root images, the more similar their estimates become. No matter the level of experience though, the annotation of minirhizotron images clearly is not objective if done by humans, which hinders comparisons between studies or even between years within long-term studies. For the training of RootDetector, we invested roughly 1300 (± 200) h annotating training images. Compared to an estimated duration of > 60,000 h for manual annotation of the roots in the two experimental setups used here (see methods section), this is an enormous step forward in efficiency.

Up to now, the manual annotation of roots in the minirhizotron images has been the bottleneck for studying root growth dynamics in high spatial and temporal resolution. Automated minirhizotrons for field studies exist, but manual analysis of the resulting images have so far prevented tapping their full potential concerning temporal resolution and replication[^33,34^]. Once trained to the given ecosystem (soil type, root morphology, etc.), the algorithm solves this limitation. While we assume that additional training is needed for high-quality analysis of images from other ecosystems, this re-training of the algorithm to other conditions should require less training data than the initial training^22^. According to our experience, we estimate that training the algorithm for data from a new experiment will roughly require 25–60 training images of 2550 × 2273 pixels, which may take approximately 150–300 h of manual annotation. This would mean that training and using the algorithm becomes less work than analyzing images by hand already after 70 images—which would be reached for 6 minirhizotron tubes with 3 image levels after only one month of weekly sampling. As minirhizotron tubes are often the least expensive part of a respective experimental set-up and as roots are highly variable in space, a high number of replicate tubes is clearly desirable. Similarly, it is known that, especially in highly productive ecosystems, fine root lifespan can be a few days or weeks only^35^ which illustrates the need for a high temporal resolution in addition to the spatial one. This goal can only be achieved if the resulting images are annotated automatically.

The RootDetector CNN reached a F1 score of 0.6044 for our field study of various wetlands. This is lower than previously published algorithms for root segmentation in rhizotron images achieved (Wang et al.^15^: F1 = 0.6479; Smith et al.^14^: F1 = 0.7; Narisetti et al.^13^F1 = 0.87). However, those studies were conducted under highly controlled conditions, often with single plant species and homogeneous mineral soils resulting in much more uniform soil and root appearance and therefore higher quality images than what can commonly be achieved under field conditions (a detailed comparison of these algorithms is shown in Table 1). Thus, to increase the understanding of root growth dynamics and their influence on ecosystem processes, we aimed to develop a tool that gives consistently accurate measures of root pixels and root length on minirhizotron images from natural plant communities, even when those are growing in organic soils consisting of plant material in varying degrees of decomposition. The poor performance of traditional automatic image analysis tools has left field ecologists with little choice but to continue analysing minirhizotron images by hand, limiting the amount of data that can be processed and ultimately our understanding of root ecology. Here, we show a very high correlation between automated annotation by RootDetector and traditional annotation by human experts. There was also no sign of changing variance in the residuals across a wide range of root lengths (Figs. 3 and 4), which further supports the conclusion that this algorithm provides a promising solution for the annotation of roots in minirhizotron images in ecological studies. As to be expected, RootDetector works best on clear and sharp images with strong contrast between roots and substrate. Just like in human analysts, quality of analysis may be disrupted by foggy images due to water condensation, daylight entering the minirhizotron at substrate surface or cavations in the substrate opening to the minirhizotrone and leaving the image out of focus. Independent of manual or automatic detection of the roots, we therefore advice to put strong emphasis on the quality of minirhizotrone imagery during sampling, as there is little to nothing that can be done to it after-the-fact. Nonetheless, RootDetector showed reasonable performance even for images with subpar quality such as the ones where human analysts differed mostly (Fig. 3). Other linear, root-like structures, such as earthworms or outer rims of water droplets, were only very seldomly misidentified as roots and the detection of the tape covers worked well. We have observed roots from 1 pixel (0.084 mm) to ~ 100 pixel (8.4 mm) width. We are not aware of any biases in detecting roots below 50 pixels (4.2 mm) width. In wider roots however, the two edges are sometimes identified as separate roots as the space between is wrongly identified as soil.

**Table 1:** Comparison of recent CNN-based root detection tools in terms of development and functionality.

	SegRoot	Adapted U-Net	faRIA	RootDetector
Reference	Wang et al.^15^	Smith et al.^14^	Narisetti et al.^13^	This study
F1 Performance Evaluation	0.6479	0.70	0.87	0.6044
Experimental setting	Greenhouse	Mesocosms	Greenhouse	Mesocosms and field
Ecosystem	Agricultural	Agricultural	Agricultural	Fen peatlands
Species	Soy	Chicory	Maize	Multispecies
Rhizotron type and -dimensions	Minirhizotron, 64 mm inner diameter	Large Rhizobox	IPK plant phenotyping system ^36^	Minirhizotron, 64 mm inner diameter
Image sampling	Circular Scanner (CI-600)	Compact Camera (Olympus Tough TG 860)	Near-Infrared Monochrome camera (UI-5200SE-M-GL, IDS	Circular scanner (CI-600)
Image dimension; resolution	2550 × 2273 at 100 dpi	3991 × 1842	2345 × 2665	2550 × 2273 at 100 dpi
Root traits	Root pixels, root length	Root pixels	75 Root traits	Root pixels, root length, root pixel turnover**
Transferability	Possible*	Possible*	Possible*	Possible**

*Possible according to source publication.

**Transfer to other ecosystems and in-depth retraining options as well as module for assessment of turnover in time series to be published soon.

## Conclusion

CNNs such as RootDetector provide a reliable and efficient method for the detection of roots in minirhizotron images. In comparison with human analysts, whose ability to detect roots varies widely, RootDetector saves resources, is objective and reproducible, and performs as well as human experts. RootDetector furthermore provides not only root pixel estimations but also root length data, which is the most commonly used metric in root ecological research and is not regularly delivered by existing CNNs. RootDetector is supplied as readily usable code on GitHub, enabling easy use by ecologists without the need of advanced programming skills. Transfer to other ecosystems or technical setups of the minirhizotrons will require re-training of the algorithm, but this is an initial and one-time investment which likely will pay off already after around 70 images, i.e. during common study length.Particularly coupled with automated minirhizotrons, this tool for automatic analysis of minirhizotron images will allow for unprecedented detail and comprehensiveness in studies of root growth dynamics, thereby answering globally important ecological and biogeochemical questions.

## Supplementary Information

Supplementary Information.

## Data Availability

GitHub repository accessible under https://github.com/ExPlEcoGreifswald/RootDetector.
