# Supplementary material for: As good as human experts in detecting plant roots in minirhizotron images but efficient and reproducible: the convolutional neural network “RootDetector”
Source: Sci Rep. 2023 Jan 25;13:1399. doi: 10.1038/s41598-023-28400-x (PMC9876992; doi:10.1038/s41598-023-28400-x)

SUPPLEMENTS

As good as human experts in detecting plant roots in minirhizotron images but efficient and reproducible: The Convolutional Neural Network "RootDetector"

Bo Peters[1*], Gesche Blume-Werry[1,2], Alexander Gillert[3], Sarah Schwieger[1,2], Uwe Freiherr von Lukas[3,4], Juergen Kreyling[1]

1: Experimental Plant Ecology, Institute of Botany and Landscape Ecology, University of Greifswald, Soldmannstraße 15, D-19489 Greifswald, Germany

2: Department of Ecology and Environmental Science, Umeå University, Umeå, Sweden

3: Fraunhofer Institute for Computer Graphics Research IGD, Rostock

4: Institute for Visual & Analytic Computing, University of Rostock

Fig. S1: Visual impression of the mesocosm experiment.

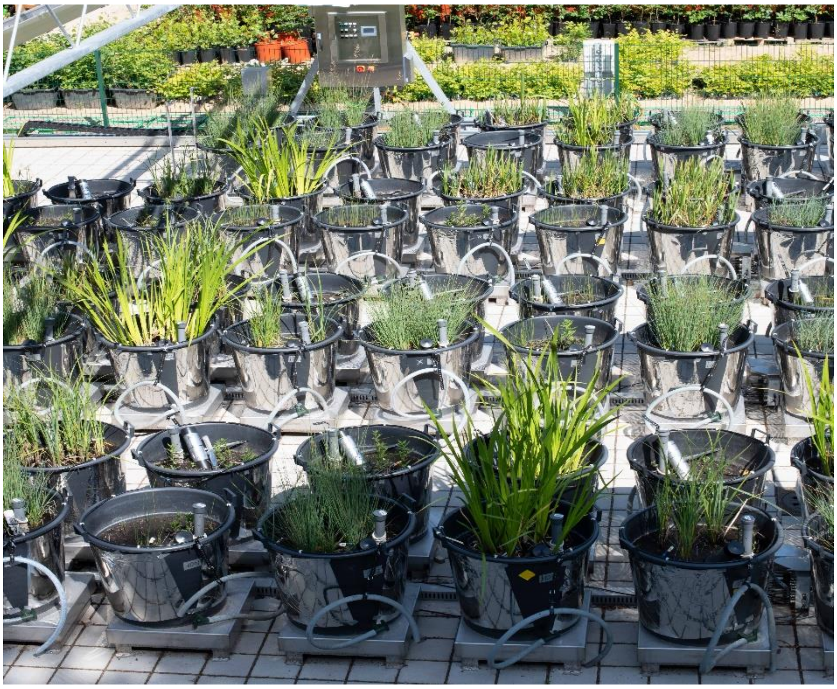

Fig. S2: Visual impressions of the different wetland types covered in the field study, including a) a sedge-dominated percolation fen, b) a coastal flood fen and c) an alder (*Alnus glutinosa*) forest. The minirhizotrons are visible in b) as silver tubes at 45° angles with their aboveground part covered by opaque silver tape preventing the entry of light.

a)

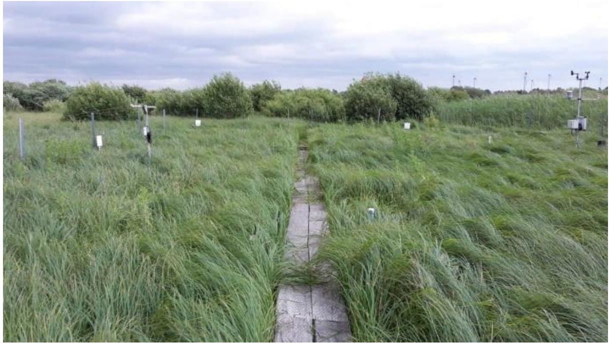

b)

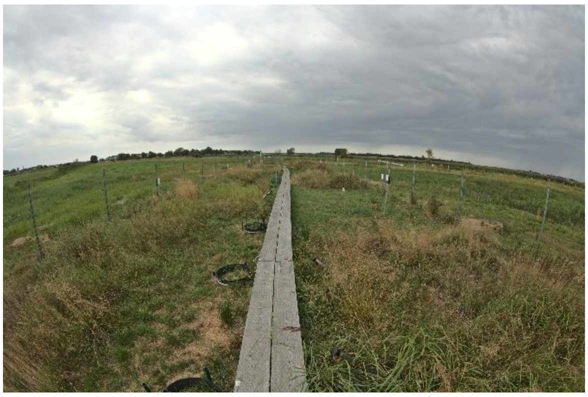

c)

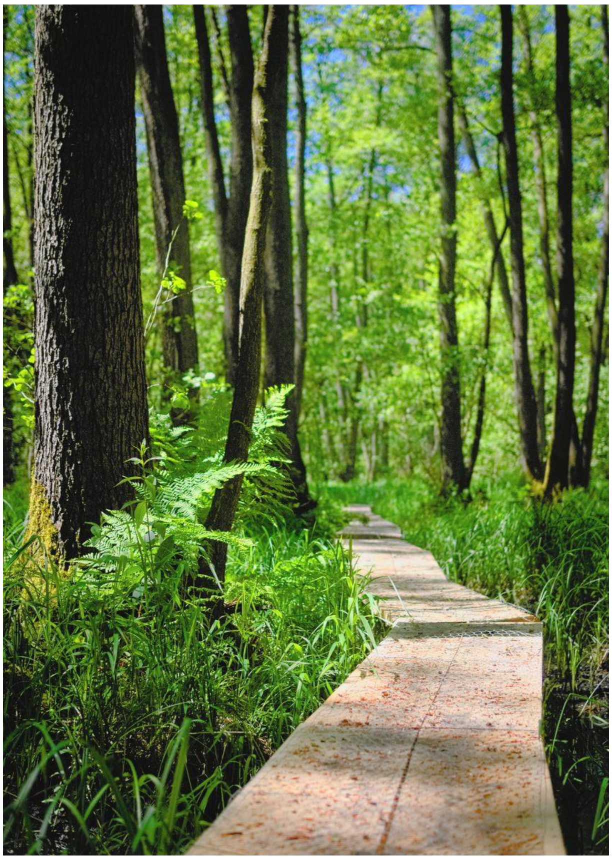

Supplement: Supplementary file 1 — Supplementary Information. [file 41598_2023_28400_MOESM1_ESM.pdf]
